# Investigation of HIFU-induced anti-tumor immunity in a murine tumor model

**DOI:** 10.1186/1479-5876-5-34

**Published:** 2007-07-11

**Authors:** Zhenlin Hu, Xiao Yi Yang, Yunbo Liu, Georgy N Sankin, Eric C Pua, Michael A Morse, H Kim Lyerly, Timothy M Clay, Pei Zhong

**Affiliations:** 1 Department of Mechanical Engineering and Materials Science, Duke University, Durham, NC 27708, USA; 2 Program in Molecular Therapeutics, Departments of Surgery, Medicine, Pathology, and Immunology, and Duke Comprehensive Cancer Center, Duke University, Durham, NC 27708, USA

## Abstract

**Background:**

High intensity focused ultrasound (HIFU) is an emerging non-invasive treatment modality for localized treatment of cancers. While current clinical strategies employ HIFU exclusively for thermal ablation of the target sites, biological responses associated with both thermal and mechanical damage from focused ultrasound have not been thoroughly investigated. In particular, endogenous danger signals from HIFU-damaged tumor cells may trigger the activation of dendritic cells. This response may play a critical role in a HIFU-elicited anti-tumor immune response which can be harnessed for more effective treatment.

**Methods:**

Mice bearing MC-38 colon adenocarcinoma tumors were treated with thermal and mechanical HIFU exposure settings in order to independently observe HIFU-induced effects on the host's immunological response. *In vivo *dendritic cell activity was assessed along with the host's response to challenge tumor growth.

**Results:**

Thermal and mechanical HIFU were found to increase CD11c+ cells 3.1-fold and 4-fold, respectively, as compared to 1.5-fold observed for DC injection alone. In addition, thermal and mechanical HIFU increased CFSE+ DC accumulation in draining lymph nodes 5-fold and 10-fold, respectively. Moreover, focused ultrasound treatments not only caused a reduction in the growth of primary tumors, with tumor volume decreasing by 85% for thermal HIFU and 43% for mechanical HIFU, but they also provided protection against subcutaneous tumor re-challenge. Further immunological assays confirmed an enhanced CTL activity and increased tumor-specific IFN-γ-secreting cells in the mice treated by focused ultrasound, with cytotoxicity induced by mechanical HIFU reaching as high as 27% at a 10:1 effector:target ratio.

**Conclusion:**

These studies present initial encouraging results confirming that focused ultrasound treatment can elicit a systemic anti-tumor immune response, and they suggest that this immunity is closely related to dendritic cell activation. Because DC activation was more pronounced when tumor cells were mechanically lysed by focused ultrasound treatment, mechanical HIFU in particular may be employed as a potential strategy in combination with subsequent thermal ablations for increasing the efficacy of HIFU cancer treatment by enhancing the host's anti-tumor immunity.

## Background

High-Intensity Focused Ultrasound (HIFU) has recently emerged as a promising non-invasive treatment modality for localized solid malignancies [1]. The fundamental principle of HIFU is to focus an acoustic beam to a small, well-defined target region. For current clinical treatment, lesion formation occurs primarily through the accumulation of heat and the subsequent coagulative necrosis at the focus, with temperatures exceeding 65°C as the target tissue absorbs the focused acoustic energy. With a typical HIFU system, the size of the induced lesion is approximately 10 mm × 1 mm. Thus, complete ablation of a tumor site is performed through progressive scanning of the tumor volume with the assistance of image guidance, such as magnetic resonance imaging or B-mode ultrasound. This treatment can be performed external to the body, provided a path devoid of air or other gaseous regions between the HIFU transducer and the target is available. In addition to the thermal mechanism, HIFU-induced tissue damage can also occur through mechanical means. With longer exposures at high pressures, HIFU can induce cavitation, the formation of microbubbles under high tensile pressure, with the resultant secondary shock wave generation and jet formation upon inertial bubble collapse [2,3]. This mechanism can cause mechanical lysis of tumor cells. Because the onset of cavitation *in vivo *is unpredictable, this method has been generally avoided in early clinical applications [2]; however, recent studies suggest that cavitation can be used to enhance HIFU-induced thermal ablation, as well as in other potential therapeutic applications such as ultrasound-mediated gene transfer and drug delivery [4-6].

Compared to conventional cancer therapy modalities, HIFU has the advantages of being noninvasive and generally well-tolerated by the patient, enabling it to be administered repetitively. Despite this advantage, several limitations in the current form of HIFU cancer therapy still exist. First, incomplete tissue necrosis, especially in large tumors, may lead to local recurrence of the tumor post-treatment. For example, about 20% local recurrence of soft tissue sarcoma has been reported [7]. This phenomenon presumably occurs due to inhomogeneities in tissue properties and heat conduction. Second, HIFU cannot be used to kill metastatic cancer cells outside the primary tumor site. In fact, distant metastasis, especially in the air-rich lung tissue, is a major cause of mortality following clinical HIFU therapy [8]. Clearly, the quality and effectiveness of HIFU cancer therapy need further improvement.

Historically, research in HIFU has been focused almost exclusively on enhancing thermal ablation efficiency with more precise control of targeting and monitoring of lesion formation, while largely ignoring the diverse range of biological responses that may be induced by HIFU. One of the most important biological consequences of HIFU treatment is the creation of a large amount of tumor antigens in the form of necrotic cells and the local release of a diverse array of endogenous danger signals from HIFU-damaged tumor cells. This biological response has the potential to stimulate an anti-tumor immune response [9,10]. However, little is known about how such significant HIFU-induced changes in the tumor microenvironment may influence the host's anti-tumor immune response. It is nevertheless interesting to note that several clinical studies have provided preliminary evidence that suggest HIFU treatment could affect the patient's immune status. For example, a marked increase in CD3^+ ^and CD4^+ ^subsets and in the CD4^+^/CD8^+ ^ratio in peripheral blood of cancer patients has been observed following HIFU treatment [8,11,12]. In addition, several animal studies have revealed encouraging evidence that suggests HIFU treatment may induce systemic anti-tumor immunity. For example, Yang et al. reported that HIFU treatment of subcutaneous murine C1300 neuroblastoma could cause a significant reduction in subsequent challenged tumor growth [13]. Despite these previous efforts, the specific immune response elicited by HIFU has not been determined using well-established immunologic assays, and the underlying mechanism is largely unknown. A strong anti-tumor immune response may help to combat residual tumor cells at the primary treatment site and to suppress distant metastasis. This potential for HIFU-elicited anti-tumor immunity clearly warrants a thorough investigation for the further development of effective cancer treatment with focused ultrasound.

Anti-tumor immunity is initiated primarily by dendritic cells (DC), which capture antigens in peripheral tumor tissues and migrate to the proximal draining lymph nodes (DLN). There, the DC present the captured antigens and sensitize antigen-specific T cells, both CD4+ helper cells and CD8+ cytotoxic T lymphocytes (CTL), to either directly attack tumor cells or to express cytokines that trigger other cells to perform this task [14,15]. To initiate an effective immune response, DC must be stimulated by "danger signals" to undergo a process of maturation. Based on Matzinger's danger theory, the maturation of DC can be induced by endogenous danger signals released from distressed or injured cells, such as those exposed to pathogens, toxins, or mechanical damage [15].

One of the biological consequences of HIFU treatment is the induction of tumor cell necrosis, resulting in the release of tumor antigens and endogenous danger signals. Such a microenvironment could attract DC to the treatment site, where they can capture the tumor antigens, mature, and migrate to adjacent draining lymph nodes to initiate an anti-tumor immune response. In an early proof-of-principle study [16], we demonstrated *in vitro *that HIFU treatments cause both thermal and mechanical necrosis. This effect leads the damaged tumor cells to release endogenous danger signals (ATP and hsp60), which could stimulate the maturation of DCs. Based on this observation, we postulate that the release of endogenous danger signals from HIFU-damaged tumor cells and the consequent activation of DCs may constitute an important mechanism for HIFU-induced anti-tumor immunity. Furthermore, we have demonstrated that the immuno-stimulatory effect of mechanically lysed tumor cells is much stronger than that of thermally ablated tumor cells, which are the predominant outcome of current HIFU cancer therapy. These findings suggest that alternative HIFU treatment strategies that promote mechanical lysis of the tumor cells (in contrast to purely thermal ablation) may elicit a stronger anti-tumor immune response.

The objective of this study is therefore three-fold: 1) to assess HIFU-induced anti-tumor immunity, 2) to investigate the underlying immunologic mechanism, and 3) to selectively produce either thermal or mechanical damage in the target tumor tissue in order to independently observe HIFU-induced effects on the host's immunological response. Subsequently, we compared the *in vivo *DC maturation and systemic anti-tumor immune response elicited by these two different HIFU treatment strategies. In-depth analysis of these systems may enable the development of improved focused ultrasound treatment strategies with increased efficacy against recurrence and metastasis. Our results confirm that HIFU treatment can indeed elicit a systemic anti-tumor immune response. Furthermore, HIFU-induced anti-tumor immunity is found to be closely related to DC activation and can be enhanced by mechanical lysis of the tumor cells.

## Methods

### Animal Model

C57BL/6 female mice, 5–8 weeks old, were purchased from the Jackson Laboratory (Bar Harbor, ME) and handled in accordance with the established animal care policy. All animal studies were approved by the Duke University Institutional Animal Care & Use Committee.

### Tumor Model

MC-38 mouse colon adenocarcinoma tumor cell line was kindly provided by Dr. Jeffrey Schlom of NCI (Bethesda, MD). EL4 Mouse lymphoma cell line was purchased from American Type Culture Collection (ATCC). Both cell lines were maintained in complete Dulbeco's modified eagle medium (DMEM), supplemented with 10% heat-inactivated fetal bovine serum (FBS) (Gibco, USA) at 37°C and 5% CO_2_. A tumor model was prepared by injecting 1 × 10^6 ^MC-38 tumor cells suspended in 50 μl of PBS subcutaneously in the left hindlimb of the C57BL/6 mice. The tumor was allowed to grow for 7–9 days to reach a maximum diameter of 8–10 mm before HIFU treatment.

### *In Vivo *HIFU Exposure System

**Figure 1:**
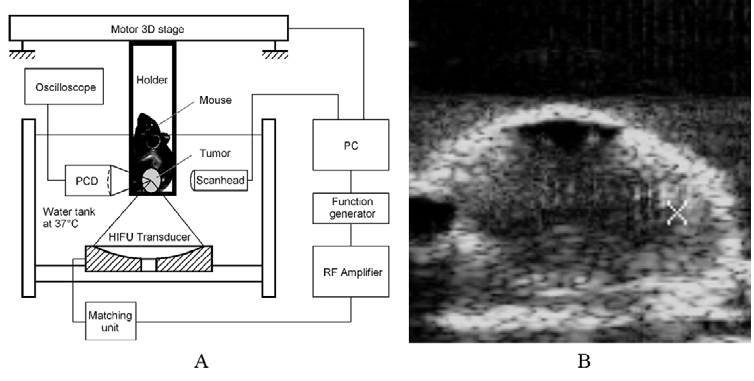
**HIFU exposure system and B-mode guidance**. (A) Diagram of the *in vivo *HIFU exposure setup. (B) Alignment of the mouse tumor with the focus of the HIFU transducer was aided by B-mode ultrasound imaging.

Figure 1A shows schematically the HIFU exposure system used in this study. The HIFU transducer (H-102, Sonic Concepts, Seattle, WA) was mounted at the bottom of a plexiglass test chamber (40 × 30 × 15 cm) filled with degassed water (37°C). This transducer has a focal length of 63 mm and was operated at 3.3 MHz (3^rd ^harmonic), driven by sinusoidal signals produced by a function generator (33120A, Agilent, Palo Alto, CA) connected in series with a 55-dB power amplifier (A150, Electronic Navigation Industries, Rochester, NY). The operation of the HIFU system was controlled by LabView programs via a GPIB board installed in a PC. During the experiment, the anesthetized tumor-bearing mouse was fixed in a custom-designed animal holder, which was connected to a 3-D positioning system driven by computer-controlled step motors (Velmex, Bloomfield NY). To facilitate alignment of the tumor to the HIFU focus, a portable ultrasound imaging system (Terason 2000, Terason, Inc., Burlington, MA) with a 5/10 MHz probe was used to provide B-mode images of the tumor cross section. The medial plane of the tumor was aligned with the focus of the HIFU transducer using this imaging system. Figure 1B shows an example of the B-mode ultrasound images of the tumor grown in the hindlimb of the mouse. As shown in the figure, the tumor outline was clearly defined, with the focus of the HIFU transducer highlighted with a cross-hair indicator. The pressure waveform and distribution in the focal plane of the HIFU transducer in water were measured and the -6 dB beam sizes along and transverse to the transducer axis were determined to be 5 × 0.6 mm [17]. Due to the beam size of the transducer, treatment of the tumor was accomplished through progressive scanning of the whole tumor volume point-by-point, translating the tumor-bearing mouse incrementally with the 3-D step motor positioning system. The tumor was scanned from the lower right corner 2 mm inside the tumor boundary, progressing from right to left with a step size of 1.0 mm. Typically, a total of 12 to 16 points were treated depending on the tumor size.

### Temperature Measurement

The temperature profile at the HIFU focus was measured by using a 0.1 mm bare-wire thermocouple (Custom designed IT-23, Physitemp Inc, Clifton, NJ) inserted into the tumor tissue. Temperature output voltage was registered in an electronically compensated isothermal terminal block (TC-2190, National Instrument) and subsequently conditioned and sampled at a rate of 60 Hz using a Data Acquisition Board (NI4351, National Instrument) controlled by a LabView program. The thermocouple embedded in the tumor was first aligned to the HIFU focus by operating the HIFU transducer at a low intensity while scanning the tumor across the acoustic field to search for the position of maximum temperature increase. Using this approach, temperature elevations inside the tumor tissue were measured for the designated HIFU exposure conditions.

### Cavitation Detection

Cavitation activities inside the tumor tissue during HIFU treatment were monitored by B-mode ultrasound imaging and passive cavitation detection (PCD). The inertial cavitation bubbles inside the tumor tissue could be identified by hyperechogenicity on the B-mode ultrasound images [18]. For PCD measurements, a 3.5-MHz focused transducer (V380, Panametrics Inc, Waltham, MA) was positioned confocally with the 3.3-MHz HIFU transducer. The data acquisition and signal processing protocols employed are similar to previously reported procedures [19]. Briefly, FFT spectrums of the acoustic emission signals emanating from HIFU-induced cavitation bubbles inside the tumor were averaged and compared at different exposure settings. RMS amplitude of the broadband noise signals for each FFT spectrum between 4.5 and 5.5 MHz was further calculated and presented in time sequence.

### Immunohistochemistry

Two days after the HIFU treatment, tumors were surgically excised, freshly frozen in Tissue-Tek O.C.T. compound (Sakura Finetek, Torrance, CA USA), and sectioned at 6 μm thickness. For immunohistochemistry, the cryostat sections were fixed in acetone and immunostained with hamster anti-mouse CD11c mAb (clone HL3, PharMingen). Subsequently, the antibody was visualized using an anti-hamster Ig HRP detection kit (Pharmingen) following the manufacturer's protocol. Finally, sections were counterstained with hematoxylin and evaluated by light microscopy.

### Generation of Murine DC

Murine DC were generated from the bone marrow of mice using established protocols [20]. Briefly, marrow from tibias and femurs of C57BL/6 mice were harvested, followed by treatment of the precursors with ammonium chloride Tris buffer for 3 min at 37°C to deplete the RBC. The precursors were plated in RPMI 1640 (Sigma, Sigma-Aldrich, St. Louis, MO)-10% FCS (GIBCO^®^, Invitrogen, Carlsbad, CA) with GM-CSF (10 ng/ml) and IL-4 (10 ng/ml) (PharMingen, San Diego, CA). Cells were plated at 1 × 10^6 ^cells/ml and incubated at 37°C and 5% CO_2_. Three days later, the floating cells (mostly granulocytes) were removed, and the adherent cells were replenished with fresh medium containing GM-CSF and IL-4. Non-adherent cells were harvested on day 6 as immature DC.

### Migration Assay of DC

For DC migration studies [21], day 6 DC were washed and labeled with 1 μM CFSE (Molecular Probes, Eugene, OR) following the manufacturer's protocol. CFSE-labeled DC were injected intratumorally 1 day after HIFU treatment. Two days after DC injection, the draining lymph nodes (LN) were harvested and processed, as described by Vremec and Shortman [22]. Briefly, inguinal LN were removed, and cell suspensions were obtained after digestion with Liberase CI and Dnase I (Roche) for 40 min at 37 °C and treatment with 50 mM EDTA for 5 min to disrupt the T cell-DC complexes. The total LN cells were counted, washed, and stained with anti-CD11 mAbs (BD PharMingen, San Diego, CA) for flow cytometry analysis. After gating on live cells, 10^6 ^events were acquired to quantify the absolute number of migrated DCs per LN (CFSE-labeled CD11c^+ ^cells).

### CTL Assays [23]

Spleens were harvested from euthanized tumor-bearing mice 10 days after HIFU treatment. Splenocytes (3 × 10^7^) were re-stimulated for 5 days *in vitro *with mitomicin C-pretreated MC-38 cells (3 × 10^6^). Re-stimulated splenocytes were used as effector cells. Cytotoxic activity against target MC-38 (specific) or EL4 (irrelevant) tumor cells was assayed at different effector:target ratios by lactate dehydrogenase (LDH) release assay according to the manufacturer's instructions (Promega). LDH release assays were chosen due to radioactivity and variability concerns with chromium release assays. For the CTL assays, three independent experiments were performed with six replicate wells included in each treatment. Cytotoxicity % was calculated as:

[experimental−culture medium backgroundmaximum LDH release−culture medium background]×100
 MathType@MTEF@5@5@+=feaafiart1ev1aaatCvAUfKttLearuWrP9MDH5MBPbIqV92AaeXatLxBI9gBaebbnrfifHhDYfgasaacH8akY=wiFfYdH8Gipec8Eeeu0xXdbba9frFj0=OqFfea0dXdd9vqai=hGuQ8kuc9pgc9s8qqaq=dirpe0xb9q8qiLsFr0=vr0=vr0dc8meaabaqaciaacaGaaeqabaqabeGadaaakeaadaWadaqaamaalaaabaGaeeyzauMaeeiEaGNaeeiCaaNaeeyzauMaeeOCaiNaeeyAaKMaeeyBa0MaeeyzauMaeeOBa4MaeeiDaqNaeeyyaeMaeeiBaWMaeyOeI0Iaee4yamMaeeyDauNaeeiBaWMaeeiDaqNaeeyDauNaeeOCaiNaeeyzauMaeeiiaaIaeeyBa0MaeeyzauMaeeizaqMaeeyAaKMaeeyDauNaeeyBa0MaeeiiaaIaeeOyaiMaeeyyaeMaee4yamMaee4AaSMaee4zaCMaeeOCaiNaee4Ba8MaeeyDauNaeeOBa4MaeeizaqgabaGaeeyBa0MaeeyyaeMaeeiEaGNaeeyAaKMaeeyBa0MaeeyDauNaeeyBa0MaeeiiaaIaeeitaWKaeeiraqKaeeisaGKaeeiiaaIaeeOCaiNaeeyzauMaeeiBaWMaeeyzauMaeeyyaeMaee4CamNaeeyzauMaeyOeI0Iaee4yamMaeeyDauNaeeiBaWMaeeiDaqNaeeyDauNaeeOCaiNaeeyzauMaeeiiaaIaeeyBa0MaeeyzauMaeeizaqMaeeyAaKMaeeyDauNaeeyBa0MaeeiiaaIaeeOyaiMaeeyyaeMaee4yamMaee4AaSMaee4zaCMaeeOCaiNaee4Ba8MaeeyDauNaeeOBa4MaeeizaqgaaaGaay5waiaaw2faaiabgEna0kabigdaXiabicdaWiabicdaWaaa@9FC0@

### ELISPOT Assay [24]

Splenocytes from mice in each group were restimulated *in vitro *by culture with mytomicin-pretreated MC-38 or EL4 tumor cells (20:1 responder-to-stimulator ratios) for 48 h. Re-stimulated splenocytes (1 × 10^6 ^cells in 100 μl medium) were then plated in 96-well nitrocellulose filter plates pre-coated with anti-mouse interferon-γ antibody (anti-IFN-γ Ab). After incubation for 24 h at 37°C and 5% CO_2_, the plates were washed with PBS, and "spots," corresponding to cytokine-producing cells, were visualized by incubation with 100 μl per well of biotinylated anti-mouse IFN-γ Ab (R&D Systems, Inc) overnight at 4°C. This was followed by incubation with 100 μl per well of streptavidin-alkaline phosphatase for 2 h at room temperature and 100 μl per well of BCIP/NBT substrate solution (ELISpot Blue Color Module, ICI Americas, Inc., Wilmington, DE) in the dark for 30 min at room temperature. IFN-γ spot-forming cells were counted using a Zeiss Axioplan II ELISPOT reader system using KS version 4.3 software (Carl Zeiss, Thornwood, N.Y.). The results were expressed as the number of spot-forming cells per 10^6 ^input cells. Overall, three independent experiments were performed with six replicate wells included in each treatment.

### Statistical Analysis

Tests of statistical significance were performed using Statview 4.1 (Abacus Concepts, Berkeley, CA). Results were acquired from the Student's *t *test using a two-tailed distribution. Statistical results are reported as *P *values.

## Results

### Temperature Increase, Cavitation, and Tissue Injury

Two different HIFU exposure conditions were employed to compare the contribution of thermal vs. mechanical damage to HIFU-induced anti-tumor immunity. For thermal HIFU, the transducer was run in continuous wave (CW) mode at a comparatively low pressure level (P^+ ^= 19.9 MPa, P^- ^= -7.7 MPa) and a short exposure time (3 s) to achieve a rapid temperature rise in the tumor tissue while limiting cavitation-mediated tissue damage. In contrast, for mechanical HIFU, the transducer was operated in burst mode with a duty cycle of 2% with a higher pressure level (P^+ ^= 34.1 MPa, P^- ^= -12.5 MPa) and a longer exposure time (30 s) to limit temperature increase while generating strong cavitation, and thus causing mechanical lysis of the tumor cells.

**Figure 2:**
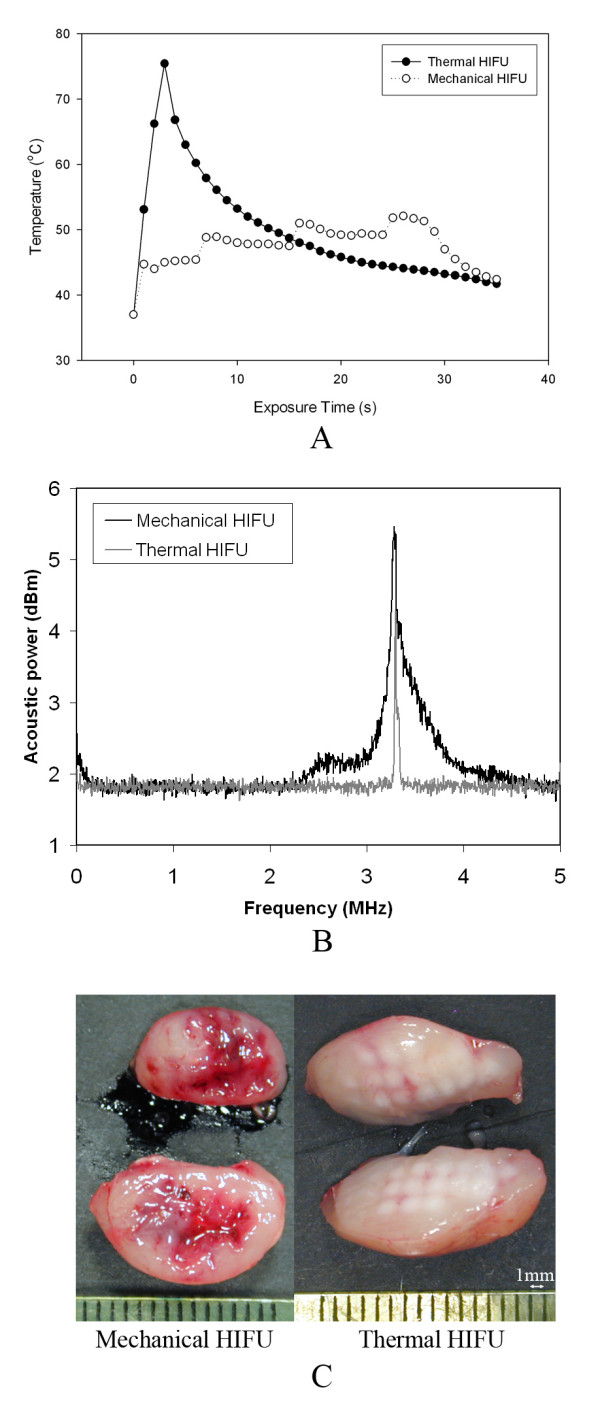
**Characteristics of thermal and mechanical HIFU treatments**. (A) *In vivo *temperature profile, (B) representative frequency spectrum of passive cavitation detection (PCD) signals, and (C) images of tumor tissue injury produced by different HIFU treatments

**Figure 3:**
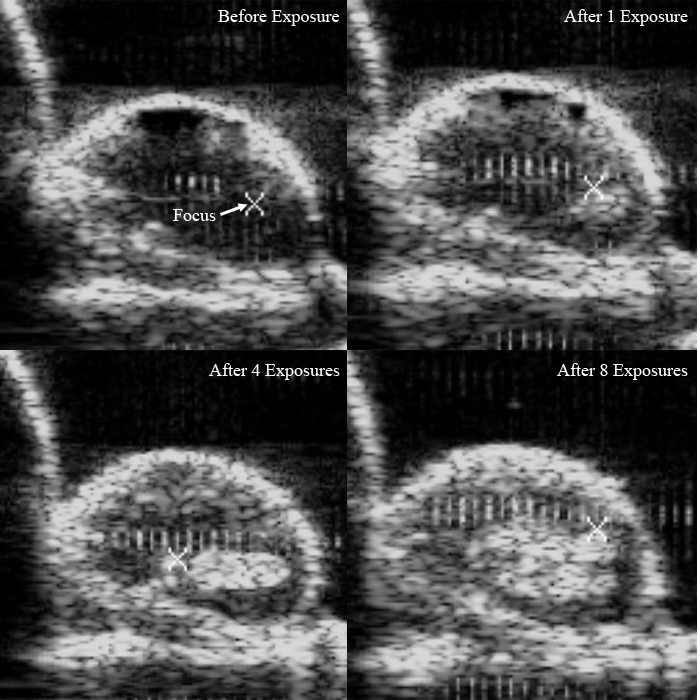
**Ultrasound imaging of HIFU-induced cavitation**. Time progression of a mechanical HIFU-exposure with B-mode ultrasound imaging. Bright hyperechoic spots generated by HIFU indicate regions of cavitation.

In thermal HIFU treatment, the temperature in the tumor tissue at the HIFU focus increased rapidly to greater than 70°C within a three second exposure, as shown in Fig. 2A. This temperature profile is representative of the clinical HIFU dosage used in cancer therapy [25,26]. Visual examination of the tumor tissue after thermal HIFU treatment revealed regular and well-defined thermal lesions (Fig. 2C). With mechanical HIFU, the focal temperature in the tumor tissue increased slowly to less than 55°C within a 30 second exposure (Fig. 2A). However, the development of bright hyperechoic regions at the HIFU focus in B-mode ultrasound images indicates cavitation activity (Fig. 3). In addition, Fig. 2B shows that mechanical HIFU exposures produced broader and higher amplitude acoustic emission signals based on passive cavitation detection. This strong cavitation activity was further confirmed by visual examination of the tumor post-treatment, revealing tumor tissue disruption (Fig. 2C). Altogether, these results suggest that a significantly higher level of cavitation activities and associated mechanical lysis was generated in the targeted tumor tissue by the mechanical HIFU exposure as compared with conventional thermal HIFU conditions.

### DC Infiltration and Migration to DLN

**Figure 4:**
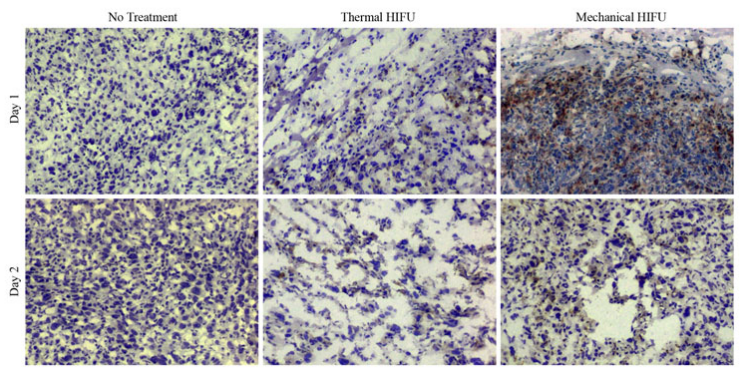
**HIFU-induced DC infiltration**. HIFU-enhanced DC infiltration into the tumor. Tumor tissue samples were collected 1–2 days after HIFU treatment. 6-μm cryostat sections were cut and stained with anti-CD11c Abs. Then the antibody was visualized using the Anti-Hamster Ig HRP detection kit. The sections were counterstained with hematoxylin.

In our previous *in vitro *study, we have demonstrated that HIFU treatment can cause the release of endogenous danger signals from damaged tumor cells and stimulate the maturation of DCs. Moreover, the immuno-stimulatory effect of mechanically lysed tumor cells was found to be much stronger than that of thermally ablated tumor cells [16]. To confirm this observation *in vivo*, we first evaluated DC infiltration into HIFU-treated tumors by immunohistochemistry. Tumor tissue samples were collected 1–2 days after HIFU treatment, and 6-μm cryostat sections were cut and stained with anti-CD11c Abs. Figure 4 shows the results of a representative experiment. In the untreated tumor, only a small amount of DC infiltration was observed, as indicated by brown regions in the image. In contrast, DC infiltration was significantly enhanced in HIFU-treated tumor tissues, especially in regions surrounding the lesions. Furthermore, mechanical HIFU treatment was found to generate more pronounced DC infiltrations than thermal HIFU treatment. The DC infiltration was most significant one day after HIFU treatment, and gradually decreased from day 2. Concomitantly, substantial enlargement of the draining lymph nodes (DLN) in HIFU-treated mice was observed on day 1, reaching maximum size between day 3 and day 4 (data not shown). These observations suggest that HIFU treatment might attract DC to the damaged regions, and, following *in situ *activation by the endogenous danger signals released from the damaged tumor cells, these infiltrated DC could migrate to DLN.

**Figure 5:**
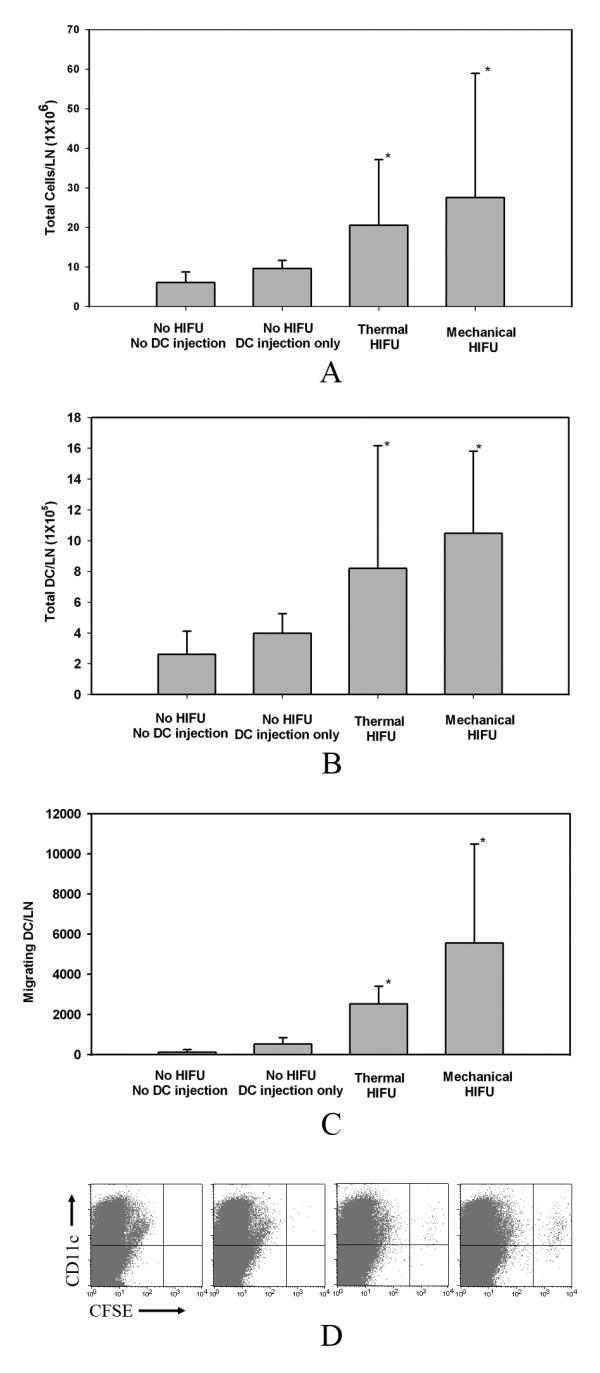
**HIFU-induced DC migration**. HIFU treatment-induced DC migration to DLN and consequent LN congestion. CFSE-labeled immature bone marrow-derived DCs were injected into tumor 1 day after HIFU treatment. The (A) total cell number, (B) total number of DC(CD11c^+ ^cells), and (C) migrating DC (CFSE^+ ^CD11c^+ ^cells) recovered in DLN on day 2 were determined by flow cytometry. Data points represent the mean ± SD for each group (n = 8). *P < 0.05 versus DC injection only control group. (D) Representative histogram illustrating the population of CFSE^+ ^CD11c^+ ^cells within the DLN of mice subjected to different treatments.

To examine this hypothesis, *in vivo *DC migration was assessed by monitoring the accumulation of CFSE-labeled DC in the regional DLN. *In vitro*-generated immature DCs were labeled with fluorescent dye CFSE and injected into the tumor one day following HIFU treatment. Draining lymph nodes were harvested 2 days after DC injection, from which single cell suspensions were prepared following an established protocol [22]. DLN cells were stained with anti-CD11c Ab, and the number of LN total cells, DC, and CD11c^+ ^and CFSE^+ ^cells were measured by FACS analysis. Consistent with the observation of DLN enlargement following HIFU treatment, a significant increase in the total cell number (Fig. 5A) and the total CD11c positive DC (Fig. 5B) in the DLN were observed. For total cell count, thermal HIFU treatment resulted in an average 3.4-fold increase from the control experiment, as opposed to an average 1.6-fold increase with DC injection alone. Furthermore, mechanical HIFU resulted in an average 4.6-fold increase over control experiments. For the total number of CD11c+ DC, thermal HIFU treatment produced a 3.1-fold increase over the average 1.5-fold increase observed with DC injection alone. Mechanical HIFU produced an average 4.0-fold increase. The results for both thermal and mechanical HIFU exhibit a statistically significant difference (P < 0.05) over those for DC injection alone, suggesting that the enlargement of DLN following HIFU treatment may result directly from the migration of mature DC to the DLN. This speculation was further verified by the detection of highly specific CFSE-labeled DC accumulation in the regional DLN. In contrast, no CFSE^+ ^DC were detected in the inguinal DLNs contra-lateral to the DC-injection site and in the non-draining axillary LN (data not shown). Quantitatively, as shown in Fig. 5C and 5D, although a modest accumulation of CFSE^+ ^DC in the DLN of the mice that received DC injection only (i.e., without HIFU treatment) was detected, about 5-fold more CFSE^+ ^DCs were detected in mice treated by the thermal HIFU. In addition, when compared with mice in the control groups, 10-fold more CFSE^+ ^DCs were detected in mice treated by the mechanical HIFU protocol. Altogether, these results demonstrate that HIFU treatment can lead to the infiltration of DC into the target tumor, whereby DC, upon maturation, will migrate and accumulate in the DLN. Comparing the two exposure conditions, mechanical HIFU treatment was found to be more effective in prompting this immunologic response.

### HIFU-Induced Systemic Anti-Tumor Response

**Figure 6:**
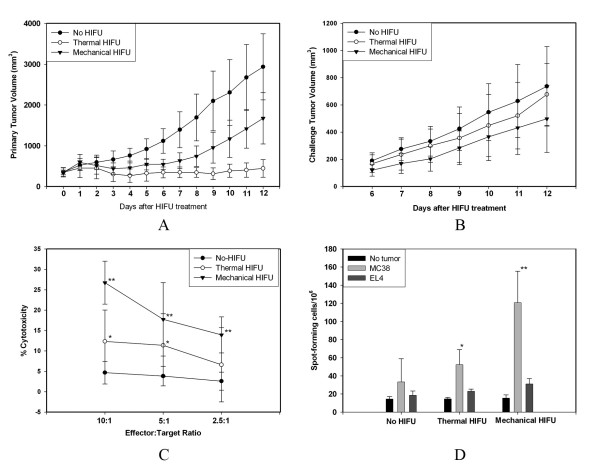
**Tumor growth and cytotoxic activity after HIFU treatment**. HIFU treatments inhibited the growth of (A) primary and (B) challenged tumors, and induced tumor-specific CTL response (C), and IFN-γ-secreting cells (D) in the spleens of treated mice. C57BL/6 mice were inoculated s.c. on right hind leg with 5 × 10^5 ^MC38 tumor cells and treated with different HIFU on day 9 of tumor inoculation. Mice were challenged with 1 × 10^6 ^MC38 tumor cells by s.c. inoculation on the left hind leg one day after HIFU treatment. Both primary and challenged tumor growth were monitored daily. Splenocytes obtained from control and treated mice 10 days after HIFU treatment were re-stimulated with mytomicin-pretreated MC-38 or EL4 tumor cells and CTL and ELISPOTS assays were performed. Results were expressed as mean value ± SD for each group (n = 8). *P < 0.05; **P < 0.001 versus non-treatment control. This experiment is representative of three experiments with consistent results.

To further assess whether HIFU treatments could induce a systemic anti-tumor immune response *in vivo*, tumor challenge experiments were performed one day following either thermal or mechanical HIFU treatment of the primary tumor by injecting 1 × 10^6 ^MC-38 cells subcutaneously in the contralateral hindlimb. The results show that both thermal and mechanical HIFU treatment impaired primary tumor growth, with thermal treatment providing more significant, long-term suppression. Figure 6A shows an average thermal HIFU-treated tumor volume 85% smaller than those left untreated, while tumors treated with mechanical HIFU were, on average, 43% smaller than those from control experiments. However, mechanical HIFU was found to have a stronger retarding effect on challenge tumor growth (Fig. 6B). To further quantify the anti-tumor immune response, we examined the CTL response following different HIFU treatments. As shown in Fig. 6C, statistically significant increases in CTL activities were detected following both thermal and mechanical HIFU treatments. Furthermore, mechanical HIFU was found to produce a much more profound increase in CTL activity than thermal HIFU with cytotoxicity reaching as high as 27% at a 10:1 effector:target ratio. No cytolytic activity of CTL against irrelevant EL4 cells was observed (data not shown), indicating that the HIFU-induced CTL was specific to the MC38 tumor. In subsequent experiments, we further evaluated whether HIFU treatment could elicit tumor-specific IFN-γ-secreting cells using ELISPOT assay. Previous studies have demonstrated that *in vitro *tumor-specific IFN-γ production by host-derived T cells correlated with systemic anti-tumor immunity *in vivo *[27]. Figure 6D shows that splenocytes retrieved on day 16 after tumor inoculation in HIFU-treated mice contained significantly more tumor-specific IFN-γ-secreting cells compared with splenocytes from the control group. This response was immunologically specific to MC38 tumor cells since it could not be detected spontaneously or in response to the stimulation of irrelevant EL4 cells. Mice treated with mechanical HIFU exhibited a 3.6-fold increase in IFN-γ-secreting cells when compared to the control group, while thermal HIFU yielded a 1.6-fold increase. These results are consistent with those from tumor challenge and CTL evaluation experiments, with mechanical HIFU affecting the tumor microenvironment much more profoundly than thermal HIFU. Taken all together, these results demonstrate that HIFU treatment can indeed induce an anti-tumor immune response, which is further enhanced by the mechanical lysis of the tumor tissues.

## Discussion

The present study was carried out to compare the anti-tumor immune response induced by two distinctly different HIFU treatment strategies *in vivo*. HIFU treatments not only can produce a reduction in the growth of the primary tumor but may also suppress distant tumor growth, indicating the induction of a systemic anti-tumor immunity. HIFU-induced anti-tumor immunity was confirmed by significantly enhanced specific CTL activity and increased tumor-specific IFN-γ-secreting cells detected in the HIFU-treated mice. In concordance with our previous *in vitro *results [16], mechanical HIFU was found to consistently induce a stronger anti-tumor immune response than thermal HIFU in all of these immunological assays.

Although the mechanisms underlying HIFU-induced anti-tumor immunity may be complex, our findings in this study suggest that HIFU can stimulate the infiltration of DCs into damaged tumor tissues. As the most effective antigen-presenting cells (APCs), DCs are initiators and modulators of the immune response. Immature DCs are distributed in peripheral tissues and continuously sample tissue antigens. In normal healthy tissues, DCs remain in a "resting state" [14], but when stimulated by an inflammatory or "danger" signal, the immature DC undergo a maturation program. This entails the down-regulation of antigen uptake capabilities, up-regulation of co-stimulatory molecules and chemokine receptors, and migration to draining LNs, where they liaise with and activate antigen-specific T cells [14]. It has been well established that the infiltration of DC into the tumor, and the subsequent migration of DC from the tumor to lymphoid organs, are critical initial steps during the induction of an anti-tumor immune response [28-30]. In this study, we have demonstrated a significantly enhanced DC infiltration into the tumor followed by pronounced DC migration to the DLN in HIFU-treated mice. In particular, in comparison to thermal HIFU, mechanical HIFU treatment was found to induce a more profound DC infiltration and subsequent migration. This important observation is further supported by the results of immune response assays showing that mechanical HIFU can induce greater tumor-specific CTL activity and an increased amount of IFN-γ-secreting T cells than thermal HIFU.

The HIFU-induced migration and maturation of DC are most likely associated with danger signals released from HIFU-damaged tumor cells. Danger signals were first postulated by Matzinger in 1994 as part of a model that suggests the immune system responds to danger, rather than to foreignness [15]. Without danger signals, as in normal healthy tissues, antigens will be presented to T cells in the absence of co-stimulation, thus resulting in tolerance. Danger signals generally consist of molecules that can be released or produced by cells undergoing stress or abnormal cell death, such as heat-shock proteins [31], nucleotides (ATP, GTP, and mammalian DNA) [32-34], uric acid [35], and chromatin protein HMBG1 (High Mobility Group B1) [36]. Some danger signals, such as HMBG1, also act as chemokines when released from necrotic cells, recruiting inflammatory cells to the injured site [36,37]. For cancer immunology, the danger signal model argues that many tumors do not appear dangerous to the immune system because they initially grow as healthy cells and do not send out distress signals to activate APCs. However, the model also predicts that *in situ *damage to the tumor cells may "set the stage" for eliciting anti-tumor immunity by providing sufficient endogenous danger signals [38]. The results from the present study reinforce this notion by demonstrating that HIFU-induced *in situ *tumor cell damage can lead to the activation of DC and is associated with a significant anti-tumor immune response. It is interesting to note that other studies have shown that chemotherapy or radiotherapy-induced tumor death may also elicit an effective anti-tumor immune response [39,40]. In addition, Schueller and colleagues have investigated immunotherapeutic strategies based on the expression of HSPs after radiofrequency ablation of tumor tissue [41].

The interaction of necrosed and dying cells with the immune system is a complicated process. The various forms of cell death, such as apoptosis, necrosis, autophagy, and mitotic catastrophe, are differentiated by particular combinations of morphological and biochemical changes, leading to the distinct release patterns of intracellular contents [42,43]. Physiologic cell death via apoptosis, in which cells maintain their membrane integrity, has long been considered to be non-immunogenic or even tolerizing [44]. In contrast, necrotic death is characterized by plasma-membrane disruption and the release of intracellular content [44]. It is therefore not surprising that necrosis is usually associated with pathology and can lead to inflammation and an immune response. However, it has been shown that both necrotic and apoptotic cells can be immunostimulatory. Still, there is speculation as to whether all forms of apoptosis are equivalent [45]. Clearly, a better understanding of the molecular mechanisms concerning different forms of cell death is needed to maximize the potential utility of endogenous danger signals. In the setting of HIFU therapy, the major form of tumor cell death is coagulative necrosis. Wu et al have performed several clinical studies evaluating the immune response induced by conventional HIFU thermal ablation, reporting a significant increase in CD4+ lymphocytes in the circulation of cancer patients after HIFU treatment [8,46]. On the other hand, HIFU may also cause mechanical rupture of the tumor cells through the action of cavitation bubbles under alternative exposure conditions, as demonstrated in this study. Our previous *in vitro *study also demonstrated that, even though many danger signals could be released from thermal HIFU-damaged tumor cells to induce DC maturation, their immunostimulatory potential was impaired due to protein denaturation at high temperatures during the rapid coagulative necrosis. In contrast, cavitation-mediated mechanical lysis of the tumor cells led to a more complete release of a diverse array of danger signals, resulting in a stronger activation of DC [16]. HIFU has definitive immunomodulatory properties and is a potential adjuvant for cancer immunotherapy, if its cell injury potential can be appropriately harnessed. The low effectiveness of current immunotherapy is most likely due, in large part, to an unfavorable tumor microenviroment for effective tumor immunity [47]. For example, tumor cells may actively produce anti-inflammatory cytokines such as TGF-β, IL-10 in order to suppress DC function and promote tolerance [48]. HIFU treatment, by injuring tumor cells and providing more effective danger signals, may overcome its inhibitory effects and turn an unfavorable tumor microenviroment into one that is conducive for DC activation. Therefore, strategies to enhance tissue necrosis in the tumor mass with HIFU before intratumoral administration of DC or cytokines should be evaluated in future studies.

## Conclusion

In the present study, our results show that while mechanical HIFU is not as effective as thermal HIFU in necrosing tumor tissue, it can induce a stronger anti-tumor response. While thermal damage produced greater suppression of tumor growth at the primary site, mechanical damage at the primary tumor site induced a stronger inhibitory effect on challenge tumor growth. In addition, mechanical HIFU caused a more profound infiltration and effective migration of dendritic cells. Further, mice treated by mechanical HIFU exhibited enhanced CTL activity and an increased population of tumor-specific IFN-γ-secreting cells over control subjects and subjects treated with thermal HIFU.

These preliminary findings demonstrate the feasibility of developing a synergistic combination of HIFU-induced thermal ablation for treating the primary tumor mass and HIFU-elicited host anti-tumor immunity for combating residual and metastatic tumor cells through mechanical lysis. Such a strategy may potentially overcome some of the primary drawbacks associated with current clinical HIFU therapy and could significantly improve the overall effectiveness of HIFU in cancer treatment. Future studies should be carried out to optimize the thermal and mechanical HIFU exposure settings and to analyze the tumor microenvironment using such strategies.

## Competing interests

The author(s) declare that they have no competing interests.

## Authors' contributions

ZH wrote the manuscript with input from the other authors. ZH, XY, YL, GN and EP performed experiments and data analysis. ZH, MM, HL, TC and PZ planned and supervised the study. All authors read and approved the final manuscript.
